# An improved data-free surrogate model for solving partial differential equations using deep neural networks

**DOI:** 10.1038/s41598-021-99037-x

**Published:** 2021-09-30

**Authors:** Xinhai Chen, Rongliang Chen, Qian Wan, Rui Xu, Jie Liu

**Affiliations:** 1 Science and Technology on Parallel and Distributed Processing Laboratory, National University of Defense Technology, Changsha, 410000 China; 2 Laboratory of Software Engineering for Complex System, National University of Defense Technology, Changsha, 410000 China; 3 Shenzhen Institutes of Advanced Technology, Chinese Academy of Sciences, Shenzhen, 518000 China

**Keywords:** Applied mathematics, Computational science, Computer science

## Abstract

Partial differential equations (PDEs) are ubiquitous in natural science and engineering problems. Traditional discrete methods for solving PDEs are usually time-consuming and labor-intensive due to the need for tedious mesh generation and numerical iterations. Recently, deep neural networks have shown new promise in cost-effective surrogate modeling because of their universal function approximation abilities. In this paper, we borrow the idea from physics-informed neural networks (PINNs) and propose an improved data-free surrogate model, DFS-Net. Specifically, we devise an attention-based neural structure containing a weighting mechanism to alleviate the problem of unstable or inaccurate predictions by PINNs. The proposed DFS-Net takes expanded spatial and temporal coordinates as the input and directly outputs the observables (quantities of interest). It approximates the PDE solution by minimizing the weighted residuals of the governing equations and data-fit terms, where no simulation or measured data are needed. The experimental results demonstrate that DFS-Net offers a good trade-off between accuracy and efficiency. It outperforms the widely used surrogate models in terms of prediction performance on different numerical benchmarks, including the Helmholtz, Klein–Gordon, and Navier–Stokes equations.

## Introduction

Numerical simulations play a vital role in the fields of scientific and engineering applications, such as aerospace, finance, civil, energy engineering, and biological engineering^1–3^. The principle of the simulation process is to solve linear/nonlinear partial differential equations (PDEs). Since the 1970s, various mesh-based numerical methods, such as finite difference (FD), finite element (FE), and finite volume (FV) methods, have been developed to solve PDE systems^4^. These methods first discretize the computational domain into mesh units and then iteratively solve the system of PDEs on each subdomain in order to yield an analysis capability for the numerical solution of the unknown functions.

However, traditional discrete methods often involve tedious meshing and iterative solving of large sparse nonlinear systems, which are computationally cumbersome on modern parallelized architectures^5–8^. Moreover, the current meshing process is still a highly specialized activity that remains in the empirical, descriptive realm of knowledge, especially for complex geometries and physical configurations^9,10^. Careful human-computer interaction is usually required to ensure a valid, high-quality mesh for the convergence of the PDE solvers. The extensive computational overhead and manual interaction limit the use of a principled PDE model for real-time analysis and optimization design. Therefore, developing a cost-effective surrogate model is desirable. An ideal model is one that takes the coordinates of some random points in the computational domain and automatically outputs the corresponding degrees of freedom of the PDE.

To fulfill this role, Raissi et al.^11,12^ employed machine learning techniques (Gaussian process and Bayesian regression) to devise functional representations for linear/nonlinear operators in physical and mathematical problems. Tartakovsky et al.^13^ presented a physics-informed machine learning approach, PICKLE, for elliptic diffusion equations. This approach uses conditional Karhunen–Lo$\grave{e}$ve expansion (cKLE) to minimize the PDE residuals and approximate the observed parameters and states. Ahalpara^14^ developed a surrogate model for solving the Korteweg–de Vries (KdV) equation using a genetic algorithm. A random forest regression model was introduced by Wang et al.^15^ to predict the Reynolds stresses in the flow over periodic hills. However, the above machine learning-based models show difficulties in generalization to different physical problems. Moreover, due to the limited approximating capacity of machine learning techniques, these models may not guarantee the desired prediction result and tend to yield an inaccurate solution for complex nonlinear PDE systems.

Deep neural networks (DNNs) are rapidly gaining more attention in many physical problems requiring intensive computing and extensive domain expertise^16–20^. DNNs utilize multiple layers of interconnected neurons to automatically learn important features from high-dimensional parameter spaces. By performing an optimization process based on the loss function, the network model brings the promise of a powerful approach to approximate the complex and nonlinear mapping relations of the input-output model. Theorems in^21,22^ prove that the universal function approximation capabilities of neural networks open a new way to obtain the latent solutions of PDE systems.

Recently, pioneering works began to explore the possibility of solving PDEs via deep neural networks. Raissi, Perdikaris, and Karniadakis^23,24^ first introduced physics-informed neural networks (PINNs) to solve forward and inverse problems involving PDEs. In PINNs, the governing equations, as well as the initial/boundary conditions, are embedded in the loss function as penalizing terms in order to constrain the space of latent solutions. Then, one trains and updates the variables (weights and biases) in the constructed network by minimizing this loss function using optimization methods such as gradient descent methods and quasi-Newton methods. After suitable training, the resulting network is able to form a new class of data-free universal function approximators that naturally encode any underlying physical laws as prior information and provide the solution to a PDE system. Although PINN-based methods appear to be straightforward, these methods usually incur difficulties in satisfying all equation residuals (especially for boundary conditions), leading to slow convergence or unstable approximation results of some local solutions.

To solve the deficiency of original PINNs, Wang et al.^25^ studied the gradient pathologies in physics-informed neural networks and introduced a gradient pathology physics-informed neural network (GP-PINN) for PDE solving. The proposed network balances the interplay between the different terms in the loss function by reweighting gradients during backpropagation training. Inspired by Galerkin methods, Sirignano and Spiliopoulos^26^ proposed a well-designed long short-term network, called the deep galerkin method (DGM), to solve high-dimensional PDEs. The DGM is trained to satisfy the differential operator and the initial/boundary conditions, thus providing an approximation to the latent solution. Their method has been successfully used in different contexts, such as approximating very high-dimensional problems arising in mathematical finance by exploiting integral representation formulas for the underlying solutions. However, one disadvantage of this method is the need for additional parameters and computational effort to obtain satisfactory solution accuracy, which leads to a significant increase in the training and prediction overhead. Lu et al.^22^ proposed deep operator networks DeepONets to learn nonlinear operators for differential equations. This network employs two subnetworks (truck and branch net) in order to extract the operator-related features from prior knowledge and then approximates the mapping relations between the input function and the unknown operator. Despite the high efficiency and flexibility, this type of method is data-dependent and supervised, which means that a labeled dataset is required for the supervised training process, therefore the quality and scale of this dataset can greatly affect the prediction performance of the underlying operator networks.

In this paper, we develop an improved data-free surrogate model, DFS-Net, to enable fast and accurate inference of PDE solutions. In the proposed methodology, we take spatial and temporal coordinates as the input and feed them into the network for training. The variables in DFS-Net are optimized by minimizing the loss function leveraging the PDE residual and data-fit terms, where no supervised simulation data are needed. Based on the observation that existing surrogate models tend to obtain unstable prediction results in different subdomains (e.g., interior or near-wall domains), we propose a weighting mechanism to calibrate the weight of input coordinates in the loss function. Moreover, we introduce an attention-based excitation block in DFS-Net to increase the approximating accuracy and accelerate the training. The experimental results show that DFS-Net can achieve a good trade-off between accuracy and efficiency. It outperforms the widely used surrogate models and yields remarkable prediction results on different benchmarks, including the Helmholtz, Klein–Gordon, and Navier–Stokes equations.

The remainder of the paper is organized as follows: in “Methodology”, we first introduce the problem setup and provide a recap of PINN for solving PDE. Next, we develop our data-free surrogate model DFS-Net. The proposed model is then applied to different PDE benchmarks to validate its robustness. The performance of DFS-Net and the comparison results with some other widely used surrogate models are discussed in “Results and discussion”. Finally, conclusions and future work are drawn in “Conclusion”.

## Methodology

### Problem setup

We begin with the description of a system of nonlinear partial differential equations (PDEs) in the following generalized form:

1 $$\begin{aligned} \frac{\partial ^ku}{\partial {t^k}}({\mathbf{x }}, \;t) \;=\; {\mathscr {D}}[u({\mathbf{x }}, \;t)], \quad \quad {\mathbf{x}} \in \Omega , \quad t \in [0,\;T], \end{aligned}$$

where the spatial domain $\Omega \in {\mathbf{R}} ^d$, ${\mathscr {D}}$ represents the differential operator, and $u({\mathbf{x}} , \;t)$ is the unknown solution we wish to solve for. The above system is subject to the well-posed boundary condition,

2 $$\begin{aligned} {\mathscr {D}}_{bc}[u({\mathbf{x }}, \;t)] \;=\; h({\mathbf{x} }, \;t), \quad \quad {\mathbf{x }} \in \partial {\Omega }, \end{aligned}$$

and the initial condition,

3 $$\begin{aligned} \frac{\partial ^lu}{\partial {t^l}}({\mathbf{x}} , \;0) \;=\; g({\mathbf{x}} ), \quad \quad l=0, \ldots ,k-1, \quad {\mathbf{x}} \in \Omega , \end{aligned}$$

where $\partial \Omega$ denotes the boundary of $\Omega$, and ${\mathscr {D}}_{bc}$ is the differential operator that imposes boundary conditions for the PDE. $h({\mathbf{x}} , \;t)$: ${\mathbf{R}} ^{d+1}$ $\mapsto$ ${\mathbf{R}}$, $g({\mathbf{x}} )$: ${\mathbf{R}} ^{d}$ $\mapsto$ ${\mathbf{R}}$ are given functions.

When a set of physical parameters and data-fit terms are given, the unknown function of interest $u({\mathbf{x}} , \;t)$ can be solved by discretizing the nonlinear/linear system using traditional numerical methods, such as the finite difference method, the finite element method, and the finite volume method. However, these methods involve tedious mesh generation and iterative solving, which heavily increases the simulation overhead.

### Deep neural network and physics-informed training

Deep neural networks have proven their powerful learning ability in many time-consuming classification- and regression-based physical applications^9,27–29^. Well-trained networks utilize multiple layers of neural units to automatically approximate complex input-output mapping from high-dimensional parameter spaces. Mathematically, a surrogate network model *F* is built to approximate the latent solution $\hat{F}$ for the underlying application:

4 $$\begin{aligned} \hat{F}({\mathbf{x}} ,\ t) \;\approx \; F(\theta _{W,b}, {\mathbf{x}} ,\ t) , \end{aligned}$$

where $\theta _{W,b}$ is a set of network parameters, including the weights and biases of the constructed network. Minimizing the mismatch between the desired solution $\hat{F}$ and the DNN-based predictions *F* is known to be a nonconvex optimization problem. A key element in the resulting problem is the training of tuning parameters on a very high dimensional parameter space. This process can be formulated as:

5 $$\begin{aligned} Loss(\theta _{W,b},{\mathbf{x}} ,\ t ) \;=\; \Vert \ \hat{F}({\mathbf{x}} ,\ t) - F(\theta _{W,b},{\mathbf{x}} ,\ t) \ \Vert _{\Omega }, \end{aligned}$$

6 $$\begin{aligned} W^*,b^* \;=\; \mathop {\arg \min }_{W,b} \; (Loss(\theta _{W,b},{\mathbf{x}} ,\ t ), \end{aligned}$$

where $\Vert \cdot \Vert$ denotes the $L^2$-norm over the domain $\Omega$. After suitable training, one can find a set of optimal or suboptimal network parameters $W^*,b^*$, such that $Loss(\theta _{W,b},{\mathbf{x}} ,\ t )$ is as close to zero as possible.

Recently, physics-informed neural networks (PINNs)^23,24,30^ have been employed to infer PDE solutions by building complex features from multiple layers of neural units via input-output relationships. In PINNs, neurons are fully connected. A loss function satisfying the PDEs [(Eqs. (1)–(3)] is employed to constrain the optimization process of the neuron parameters. Let ${(x_n^r,\ t_n^r)}^{n=N_r}_{n=1}$ be a preselected set of spatial and temporal points inside the solution domain $\Omega$. ${(x_n^b,\ t_n^b)}^{n=N_b}_{n=1}$ and ${(x_n^i,\ t_n^i)}^{n=N_i}_{n=1}$ denote the preselected point data sampled from the boundary and initial condition, respectively. The loss function of PINN is formulated as:

7 $$\begin{aligned} Loss(\theta _{W,b},{\mathbf{x}} ,\ t ) \;=&\; \frac{1}{N_r}\sum ^{N_r}_{n=1}|\frac{\partial ^ku}{\partial {t^k}}({\mathbf{x}} _n^r, \;t_n^r) - {\mathscr {D}}[u({\mathbf{x}} _n^r, \;t_n^r)]|^2 + \frac{1}{N_b}\sum ^{N_b}_{n=1}|{\mathscr {D}}_{bc}[u({\mathbf{x}} _n^b, \;t_n^b)] - h({\mathbf{x}} _n^b, \;t_n^b)|^2 \\&+ \frac{1}{N_i}\sum ^{N_i}_{n=1}|\frac{\partial ^lu}{\partial {t^l}}({\mathbf{x}} _n^i, \;0) - g({\mathbf{x}} _n^i)|^2,  \end{aligned}$$

Given a specific neural network architecture, the PINN can be viewed collectively as a function of the input data and the parameters $$\theta _{W,b}$$. It maps the time *t*, spatial coordinates $$x $$, and variables to the quantities of interest, e.g., the velocity field *u* or pressure field *p*, thus allowing a data-free PDE “solver” that does not require meshing or numerical iteration. If the physics-based loss function $$Loss(\theta _{W,b},{\mathbf{x}} ,\ t )$$ becomes identically zero, the output predictions will exactly satisfy the underlying PDE system. However, experimental results in^31,32^ indicate that PINN shows difficulties in fitting all equation residuals and fails to guarantee a stable approximation of the solution. This may lead to serious errors in the PINN and return incorrect predictions, especially in the near-wall domains.

### DFS-Net: a data-free surrogate model for solving PDEs

Following the original works of PINN, we propose an improved deep neural network DFS-Net to tackle the aforementioned challenges.

**Figure 1:**
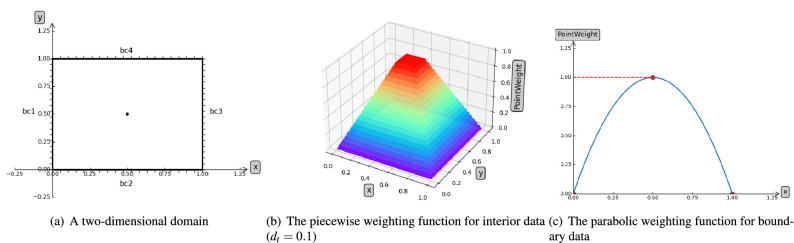
The weighting mechanism used in the proposed DFS-Net.

To alleviate the problem of unstable predictions in different subdomains and obtain more accurate results, we first introduce a weighting mechanism in DFS-Net. The key idea is to associate the weights of each training point with its coordinates in the computational domain. Through trial and observation, we found that the center points are more informative than the edge points. Based on this observation, we set the point weighting mechanism with a function that gives more weight to these “pivotal” points. Taking a 2-D domain as an example (see Fig. 1), for training points sampled inside the domain $\Omega$, we use a piecewise function $\omega$ to give different weights to points in different domains:

8 $$\begin{aligned} \omega (p) = \left\{ \begin{aligned}&\frac{1}{0.5-d_t}d_l, \quad if \quad d_l > 0.5 - d_t \\&1, \quad \quad \quad \quad else \end{aligned} \quad ,p \in \Omega , \right. \end{aligned}$$

where $d_l$ is the shortest distance of the weighted point from boundaries (bc1-bc4), and $d_t$ is an empirical parameter controlling the scope of the weighting area.

For the boundary data, $\omega$ is defined as a parabolic function describing weight characteristics (see Fig. 1c), where the midpoint has a weight of 1 and the endpoints have a weight of 0.

9 $$\begin{aligned} \omega (p) = \left\{ \begin{aligned}&1 - y^2, \quad p \in \partial \Omega _{1,3}, \\&1 - x^2, \quad p \in \partial \Omega _{2,4}. \end{aligned} \right. \end{aligned}$$

In this way, DFS-Net is able to better balance the contribution of training points sampled from different subdomains (e.g., interior or boundary points) and accelerate the loss convergence. Moreover, this mechanism could also eliminate the potential singular values caused by discontinuities or sudden changes in the boundary conditions, thus allowing us to achieve better accuracy.

**Figure 2:**
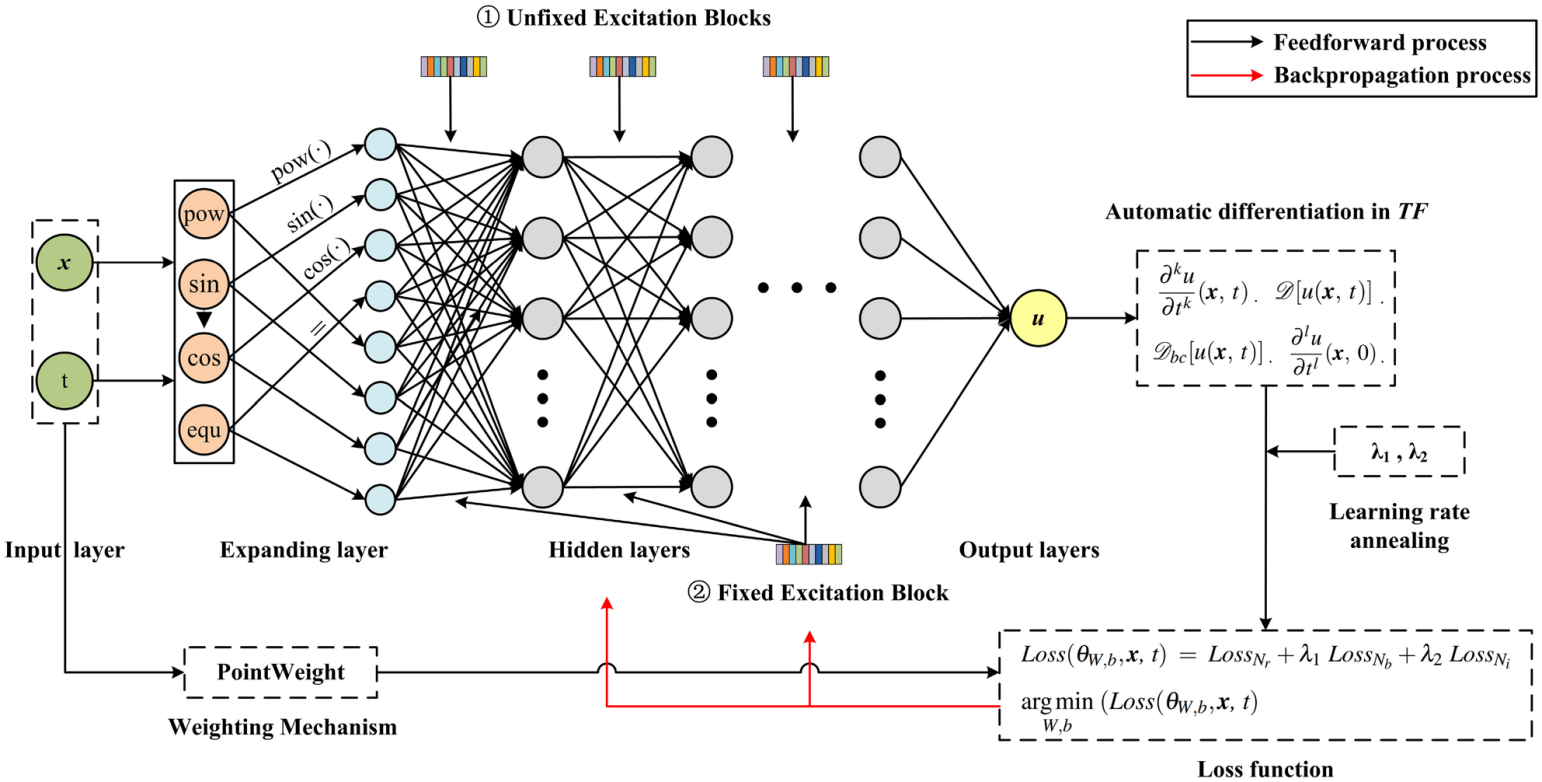
The overall pipeline of the proposed DFS-Net.

We now introduce the overall pipeline of the proposed DFS-Net. Instead of employing a very deep neural network, DFS-Net uses a lightweight structure to learn solution-related features from the space-time input. As depicted in Fig. 2, we first introduce a linear expanding layer as a data enhancement in DFS-Net. This layer defines a mapping from the input layer $z_{in}$ $\in$ $R ^2$ to the output $z_{1}$ $\in$ $R ^8$:

10 $$\begin{aligned} z_{in}[(x,t)] \mapsto z_{1}[pow(x,t),sin(x,t),cos(x,t),(x,t)], \end{aligned}$$

where $pow(\cdot )$ computes the square of the (*x*, *t*) element-wise, and $sin(\cdot )$ and $cos(\cdot )$ compute the sine and cosine of (*x*, *t*), respectively.

The affine transformation in the expanding layer can make the input $(x ,t)$ better suited for nonlinear partial differential function approximation, thereby capturing the complex high-dimensional features inherent in conservation laws. Our experiments show that the introduced expanding layer improves the performance of neural network-based surrogate models compared with no expansion, albeit leading to a higher computational cost at the beginning of the training phase.

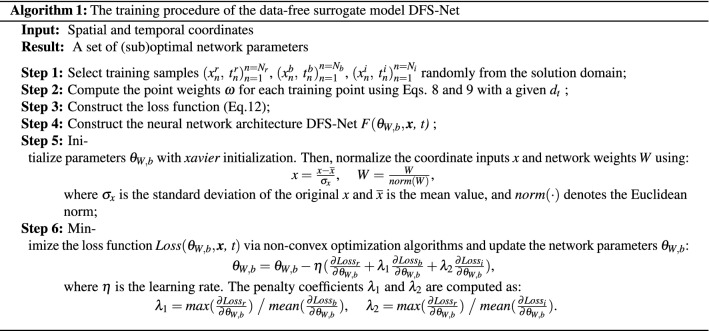



After expansion, a series of hidden layers are employed to extract the features of interest from the expanded spatial and temporal coordinates. In these hidden layers, the neurons of adjacent layers are fully connected, and each hidden layer of the network receives an output from the previous layer. Inspired by^33^, we introduce one-dimensional excitation blocks to further increase the approximating ability of DFS-Net. An excitation block is a computational unit built upon a transformation between two layers. It takes the output of the last hidden layer as the input and produces a collection of per-channel modulation weights, which can be regarded as a simple self-gating mechanism. The affine transformation in each hidden layer is computed as:

11 $$\begin{aligned} z_l = \sigma (W_l z_{l-1} + b_l) \odot W_{excitation}, \end{aligned}$$

where the subscript *l* denotes the index of the hidden layer, and $W_l$ and $b_l$ are the weight matrix and bias vector in layer *l*, respectively. The element-wise activation function $\sigma$ is applied to the transformed vector $(W_l z_{l-1} + b_l)$, for which a number of options can be chosen, e.g., *sigmoids*, rectified linear units (ReLU), and *tanh* functions^34^. $\odot$ represents a point-wise multiplication operator. $W_{excitation}$ is the weight matrix provided by the excitation block. In DFS-Net, we provide two excitation modes: (1) fixed excitation mode and (2) unfixed excitation mode. Among them, the fixed excitation mode adaptively recalibrates the per-channel feature responses by explicitly modeling interdependencies between the channels using a shared excitation block for each hidden layer. The unfixed excitation mode employs different excitation blocks for different hidden layers. Both modes help DFS-Net reweight channel attention without requiring additional supervision, where the fixed block mode induces a relatively small computational and memory overhead.

The detailed training procedure of the data-free surrogate model DFS-Net is shown in Algorithm 1. In our work, we consider the spatial and temporal coordinates as inputs. We first randomly select the training data from the solution domain. One can also sample training data uniformly depending upon space (time) scales or using other randomized designs, such as Latin hypercube sampling strategy and a truncated Gaussian distribution^23^. Then, we adopt the weighting mechanism to compute weights for each set of preselected training data, aimed at alleviating the prediction inaccuracy in the near-wall domains.

In step 3, we construct the loss function corresponding to the underlying PDE system. The loss function is used to constrain the DFS-Net, such that the conservation laws, boundary conditions, and initial conditions are satisfied at each iteration of subsequent training. The loss function $Loss(\theta _{W,b},{\mathbf{x}} ,\ t )$ of DFS-Net for solving PDEs is defined as follows:

12 $$ \begin{aligned}&Loss(\theta _{W,b},{\mathbf{x}} ,\ t ) \;=\; Loss_{N_r} + \lambda _1\ Loss_{N_b} + \lambda _2\ Loss_{N_i}, \\&Loss_{N_r} = \frac{1}{N_r}\sum ^{N_r}_{n=1}\omega _n \cdot |\frac{\partial ^ku}{\partial {t^k}}({\mathbf{x}} _n^r, \;t_n^r) - {\mathscr {D}}[u({\mathbf{x}} _n^r, \;t_n^r)]|^2, \quad \quad Loss_{N_b} = \frac{1}{N_b}\sum ^{N_r}_{n=1}\omega _n \cdot |{\mathscr {D}}_{bc}[u({\mathbf{x}} _n^b, \;t_n^b)] - h({\mathbf{x}} _n^b, \;t_n^b)|^2, \\&Loss_{N_i} = \frac{1}{N_i}\sum ^{N_i}_{n=1}\omega _n \cdot |\frac{\partial ^lu}{\partial {t^l}}({\mathbf{x}} _n^i, \;0) - g({\mathbf{x}} _n^i)|^2, \end{aligned} $$

where $Loss_{N_r}$, $Loss_{N_b}$ and $Loss_{N_i}$ represent the loss terms corresponding to the residual of the governing equation, the boundary condition, and the initial condition, respectively. $N_r$, $N_b$ and $N_i$ denote the number of preselected point samples for different loss terms. $\omega _n$ is the weight of the *n*-th point calculated by the weighting mechanism. $\lambda _1$ and $\lambda _2$ are penalty coefficients introduced by the learning rate annealing method, which work as a dynamic penalty strategy to overcome the imbalance contribution of the governing equation term and the boundary/initial condition terms in $Loss(\theta _{W,b},{\mathbf{x}} ,\ t )$. The balanced interplay between different terms can help DFS-Net better learn the rule-based algorithm from the PDE systems and accelerate convergence during training. In our experiments, $\lambda _1$ and $\lambda _2$ are initialized by 10 and are updated every 10 gradient descent steps.

After constructing the network structure (Step 4) and initializing the network parameters (Step 5), we feed the preselected data into DFS-Net for feedforward training. The input signals are passed between all hidden layers (with activation functions) and converted into high-level features. During the backpropagation process, the loss function concludes the partial derivatives of the layer outputs with respect to the variables. The network variables (weights, biases, and $\lambda$) are optimized via non-convex optimization algorithms (e.g., stochastic gradient descent or quasi-Newton) to minimize $Loss(\theta _{W,b},{\mathbf{x}} ,\ t )$. The optimization process stops after converging to a local optimum. Thereafter, the well-trained DFS-Net with a set of (sub)optimal network parameters can be used as a black box to rapidly compute the prediction solution for any given input vector (coordinates), such as the velocity, temperature, or pressure field. Since this feedforward prediction procedure only involves a few matrix multiplications, the computational cost for the prediction can be neglected compared to that of a traditional numerical simulation.

### Training

The activation functions play an important role in neural network training. They perform a nonlinear transformation to the output of each hidden layer, making it possible for neurons to approximate complex patterns. The *swish* activation function is a widely used nonlinear mapping function for deep neural networks, which is defined as:

13 $$\begin{aligned} f(x) = x \cdot sigmoid(\beta x), \end{aligned}$$

Previous studies show that *swish* is less prone to the vanishing and exploding gradient problem^35^. In DFS-Net, we use the *swish* function with $\beta$ = 10 for activation in each hidden layer, except for the last layer, where a linear activation function is used.

For the non-convex optimization algorithm, we combine the Adam and L-BFGS-B optimizers^36^ to minimize the loss function. We first apply the Adam optimizer for stochastic gradient descent training and then employ the L-BFGS-B optimizer to finetune the results. During the Adam-based training, the optimizer randomly samples a subset of data (called a mini-batch) from the training set to calculate the direction of the gradient at each iteration. In our work, the initial learning rate is $1\times 10^{-4}$ and decays 0.9 every 1000 epochs (iterations). The mini-batch size is 128, and the number of training epochs is $1\times 10^{4}$ for Adam-based training. L-BFGS-B is a limited-memory quasi-Newton optimizer for bound-constrained optimization. It is known to work very well at escaping from local optima during network training and requires little tuning. For L-BFGS-B training, the input training set is 1280 points that are randomly sampled from the solution domain. We set the stopping criterion of L-BFGS-B to $sys.float\_info.min$, which is the minimum float value in Python^37^.

For all test cases, we trained the DFS-Net on Intel Intel(R) Xeon(R) Gold 6150 CPUs. The partial differential operators in governing equations are computed using “tf.gradients()” based on the chain rule and automatic differentiation in TensorFlow 1.15.0^38,39^. During training, the random seeds for TensorFlow and Numpy^37^ are set to 666 to ensure the reproducibility of the experimental results.

## Results and discussion

In this section, we study and compare the performance of DFS-Net with some other widely used DNN-based surrogate models for PDE solving, including PINN^23^, PINN with the learning rate annealing algorithm^25^, DGM^26^, and GP-PINN^25^. To evaluate the prediction accuracy of different models, we use the relative $L^2$-error criterion, which is defined as:

14 $$\begin{aligned} L^2-error = \frac{\Vert u_{ref} - u_{pred} \Vert _2}{\Vert u_{ref} \Vert _2}, \end{aligned}$$

where $u_{ref}$ denotes the reference solution given by the analytical solution or high-fidelity DNS numerical results, and $u_{pred}$ denotes the predicted solution obtained by surrogate models.

### Helmholtz equation

In the first test case, we use the two-dimensional Helmholtz equation as a benchmark to investigate the function approximation capability of the proposed methodology. This equation is one of the fundamental PDEs arising in various fields, such as acoustics, electromagnetism, and elastic mechanics^40^. The two-dimensional Helmholtz equation we used is given by:

15 $$\begin{aligned} \Delta u(x,y)+k^2u(x,y) = q(x,y), \quad \quad (x,y) \in \Omega , \end{aligned}$$

16 $$\begin{aligned} u(x,y)=h(x,y), \quad \quad (x,y) \in \partial \Omega , \end{aligned}$$

where $\Delta$ denotes the Laplace operator, $\Omega \in [0,1]\times [0,1]$, and *q*(*x*, *y*) is a source term given by:

17 $$\begin{aligned} \begin{aligned} q(x, y)=&-\left( \alpha _1 \pi \right) ^{2} \sin \left( \alpha _1 \pi x\right) \sin \left( \alpha _2 \pi y\right) -\left( \alpha _2 \pi \right) ^{2} \sin \left( \alpha _1 \pi x\right) \sin \left( \alpha _2 \pi y\right) + k^{2} \sin \left( \alpha _1 \pi x\right) \sin \left( \alpha _2 \pi y\right) , \end{aligned} \end{aligned}$$

To obtain the predicted solution of Eqs. (15 and 16), we formulate the following loss function to guide the subsequent training:

18 $$\begin{aligned} \begin{aligned} Loss(\theta _{W,b},x,y) \;&=\; \frac{1}{N_r}\sum ^{N_r}_{n=1}\omega _n \cdot |\Delta u(x,y)+k^2u(x,y) - q(x,y)|^2 + \frac{1}{N_b}\sum ^{N_b}_{n=1}\omega _n \cdot |u(x,y)-h(x,y)|^2. \end{aligned} \end{aligned}$$

Note that the governing equation and boundary condition are embedded in the loss terms as constraints. *h*(*x*, *y*) is computed by the analytical solution (k = 1) given by:

19 $$\begin{aligned} u_{ref} = \sin \left( \alpha _1 \pi x\right) \sin \left( \alpha _2 \pi y\right) . \end{aligned}$$

**Figure 3:**
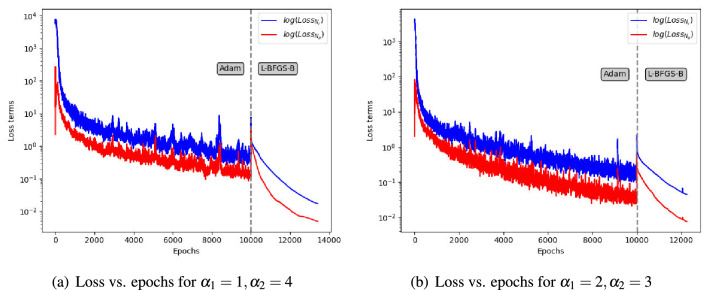
The convergence of DFS-Net (on the *log* scale) on the Helmholtz equation. The Adam optimizer is used before the vertical dashed line, and the L-BFGS-B optimizer is used afterwards.

In this case, we construct a three-hidden-layer DFS-Net with 50 neural units per layer to find the optimal network parameters for which the suitably defined loss function (Eq. 18) is minimized. We conduct experiments on two different equation settings: (1) $\alpha _1=1$ and $\alpha _2=4$ and (2) $\alpha _1=2$ and $\alpha _2=3$. Figure 3 depicts the convergence of DFS-Net on these two Helmholtz benchmarks. From the variation curves of the loss value, we can observe that applying a two-step optimization (Adam and L-BFGS-B) is robust for the DFS-Net. During the Adam training phase, the value of the loss terms ($Loss_{N_r}$ and $Loss_{N_b}$) decreases as the learning rate decays with the increase in the epoch. For the L-BFGS-B phase, DFS-Net rapidly converges after 3417 epochs (for $\alpha _1=1$ and $\alpha _2=4$) and 2251 epochs (for $\alpha _1=2$ and $\alpha _2=3$), respectively.

**Figure 4:**
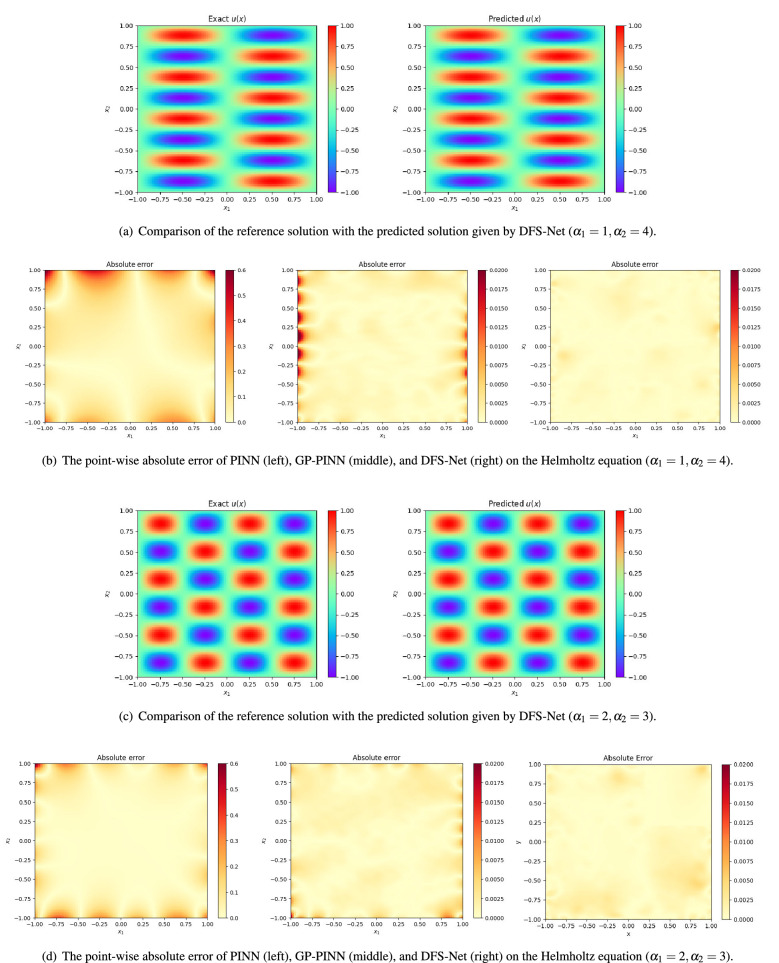
Performance of different surrogate models on the Helmholtz benchmarks.

In Fig. 4, we compare the predicted solution $u_{pred}$ with the reference solution $u_{ref}$ and report the point-wise absolute error between them. It is evident that the DFS-Net based approximation does a good job at fitting the governing equation and boundary conditions on Helmholtz benchmarks. In particular, the introduced point weighting mechanism and excitation blocks in DFS-Net effectively alleviate the problem of unstable predictions by PINNs. Compared with the absolute error of PINN and GP-PINN depicted in Fig. 4b and d, we can clearly see the advantage of the proposed DFS-Net, that is, the prediction solution is more accurate, especially in subdomains near the boundaries.

In Table 1, we compare the DFS-Nets (fixed excitation mode DFS-Net$_{fix}$ and unfixed excitation mode DFS-Net$_{unfix}$) against the other surrogate models. To ensure fairness in the comparison of different models, we set the number of hidden layers of all models to $3 \times 50$ and use the same hyperparameter settings. The experimental results show that DFS-Nets outperform the existing neural network-based solvers. When $\alpha _1=1, \alpha _2=4$, DFS-Net$_{fix}$ achieves an average $L^2$-error of 3.27e−03 in this case, while DFS-Net$_{unfix}$ yields 1.48e−03. The prediction errors of DFS-Net are approximately two orders of magnitude lower than those of PINN and DGM and one order of magnitude lower than those of PINN-anneal. Similar results can be seen in Table 2, where $\alpha _1=2$ and $\alpha _2=3$. We can see that the proposed DFS-Nets are able to better extract the solution-related features inherent in conservation laws compared to other models, and DFS-Net$_{unfix}$ achieves the best performance of 2.95e−03 on this benchmark.

**Table 1:** Comparison of the relative $L^2$-error of different neural network-based surrogate models on the Helmholtz equation ($\alpha _1=1,\alpha _2=4$).

Surrogate model^a^	Accuracy (${L}^{2}$-error)	Training time (ms)^b^
DGM	7.14e−01	44.12
PINN	2.27e−01	4.60
PINN-anneal	1.83e−02	5.41
GP-PINN	5.59e−03	12.28
DFS-Net$_{fix}$	3.27e−03	10.11^c^
DFS-Net$_{unfix}$	1.48e−03	10.66^d^

^a^All models consists of three hidden layers with 50 neurons in each layer.

^b^The training time for each Adam epoch.

^c^ The training time of DFS-Net$_{fix}$ for each L-BFGS-B epoch is 22.01 ms.

^d^ The training time of DFS-Net$_{unfix}$ for each L-BFGS-B epoch is 23.88 ms.

**Table 2:** Comparison of the relative $L^2$-error of different neural network-based surrogate models on the Helmholtz equation ($\alpha _1=2,\alpha _2=3$).

Surrogate model^a^	Accuracy ($L^2$-error)	Training time (ms)^b^
DGM	6.21e−01	43.82
PINN	1.37e−01	4.57
PINN-anneal	2.95e−02	5.34
GP-PINN	3.48e−03	12.12
DFS-Net$_{fix}$	3.14e−03	10.08^c^
DFS-Net$_{unfix}$	2.95e−03	10.68^d^

^a^All models consists of three hidden layers with 50 neurons in each layer.

^b^ The training time for each Adam epoch.

^c^The training time of DFS-Net$_{fix}$ for each L-BFGS-B epoch is 22.00 ms.

^d^ The training time of DFS-Net$_{unfix}$ for each L-BFGS-B epoch is 23.86 ms.

The training time column of Table 1 records the time overhead required to complete each training epoch. It is clear that the proposed model achieves a good trade−off between accuracy and efficiency. DFS-Net$_{fix}$ with a shared excitation block takes approximately 10 ms for one Adam epoch and 22 ms for one L-BFGS-B epoch, while DFS-Net$_{unfix}$ with unfixed excitation blocks leads to a relatively higher computational cost. The total training time required for DFS-Net to achieve the best prediction is 188.19 s and 150.64 s for two different modes, while the total time for PINN, PINN-anneal and GP-PINN are 184.0, 216.4, and 491.2, respectively.

### Klein–Gordon equation

Here, we use DFS-Net to simulate the time−dependent Klein–Gordon equation. This equation is a second-order nonlinear PDE closely related to many scientific fields, such as quantum, solid-state, and condensed matter physics^41^. The initial boundary value problem of the one−dimensional Klein–Gordon equation is given by:

20 $$\begin{aligned} \frac{\partial ^2u(x,t)}{\partial t^2} - \frac{\partial ^2u(x,t)}{\partial x^2} + u(x,t)^3 = q(x,t), \quad (x,t) \in \Omega \times [0,T], \end{aligned}$$

21 $$\begin{aligned} u(x,t)=h(x,t), \quad \quad (x,t) \in \partial \Omega \times [0,T], \end{aligned}$$

22 $$\begin{aligned} u(x,0)=g_1(x), \quad \quad x \in \Omega ,\; t=0, \end{aligned}$$

23 $$\begin{aligned} \frac{\partial u(x,0)}{\partial t}=g_2(x), \quad \quad x \in \Omega , \; t=0, \end{aligned}$$

where the computation domain $\Omega \in [0,1]$ and $T=1$. The boundary conditions *h*(*x*, *t*), initial conditions ($g_1(x),g_2(x)$), and forcing term *q*(*x*, *t*) are extracted from the analytical solution given by:

24 $$\begin{aligned} u(x, t)=x \cos (5 \pi t)+(x t)^{3}. \end{aligned}$$

A composite loss function that includes the governing equation term, boundary condition term, and initial condition term for this benchmark is formulated as follows:

25 $$\begin{aligned} \begin{aligned} Loss(\theta _{W,b},x,y) \;&=\; \frac{1}{N_r}\sum ^{N_r}_{n=1}\omega _n \cdot |\frac{\partial ^2u(x,t)}{\partial t^2} - \frac{\partial ^2u(x,t)}{\partial x^2} + u(x,t)^3 - q(x,t)|^2 + \frac{1}{N_b}\sum ^{N_b}_{n=1}\omega _n \cdot |u(x,t)-h(x,t)|^2 \\& \quad + \frac{1}{N_i}\sum ^{N_i}_{n=1}\omega _n \cdot |u(x,0)-g_1(x)|^2 + \frac{1}{N_i}\sum ^{N_i}_{n=1}\omega _n \cdot |\frac{\partial u(x,0)}{\partial t}-g_2(x)|^2. \end{aligned} \end{aligned}$$

The DFS-Net used in this case consists of five hidden layers with 50 neural units in each layer. During the DFS-Net training process, we seek to find the minimum loss value by tuning the network parameters. For this purpose, we use the chain rule to back-propagate derivatives from the output layer to the inputs and update the weights, biases, and $\lambda$. After offline training, this DNN-based surrogate model is expected to provide a rapid online prediction of observables with a set of optimal parameters $\theta$.

**Figure 5:**
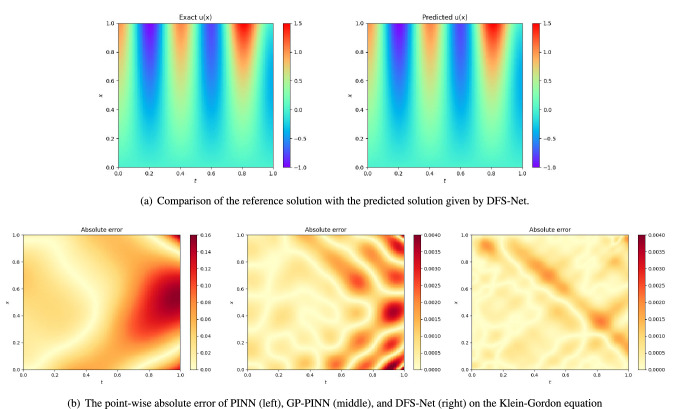
Performance of different surrogate models on the Klein–Gordon equation.

In Fig. 5a, we present the comparison results of the reference solution and the predicted solution given by DFS-Net. We also summarize the pointwise absolute error of different surrogate models on the Klein–Gordon equation in Fig. 5b. As expected, DFS-Net presents good agreement with the reference solution and achieves the smallest pointwise absolute error in the solution domain of this problem.

Table 3 provides a more detailed evaluation of the $L^2$-error for different models. As shown in Table 3, both modes of DFS-Net are able to yield solutions with a high accuracy. The network with unfixed excitation blocks (DFS-Net$_{unfix}$) achieves the best performance of 1.45e−03 at the end of 13775 epochs (10000 epochs of Adam-based training and 3775 epochs of L-BFGS-B-based training). We can also observe that by introducing the learning rate annealing method, the DNN-based surrogate models can obtain more robust prediction results (corresponding to model PINN-anneal, GP-PINN, and DFS-Nets). In contrast, PINN and DGM fail to yield the desired prediction accuracy, leaning to $L^2$-errors of 1.38e−01 and 2.09e−01, respectively.

**Table 3:** Comparison of the relative $L^2$-error of different neural network-based surrogate models on the Klein–Gordon equation.

Surrogate model	Accuracy ($L^2$-error)	Training time (ms)^a^
DGM	2.09e−01	50.74
PINN	1.38e−01	6.01
PINN-anneal	8.71e−03	7.19
GP-PINN	2.57e−03	21.88
DFS-Net$_{fix}$	2.29e−03	17.73^b^
DFS-Net$_{unfix}$	1.45e−03	19.36^c^

^a^ The training time for each Adam epoch.

^b^ The training time of DFS-Net$_{fix}$ for each L-BFGS-B epoch is 41.14 ms.

^c^ The training time of DFS-Net$_{unfix}$ for each L-BFGS-B epoch is 44.44 ms.

**Figure 6:**
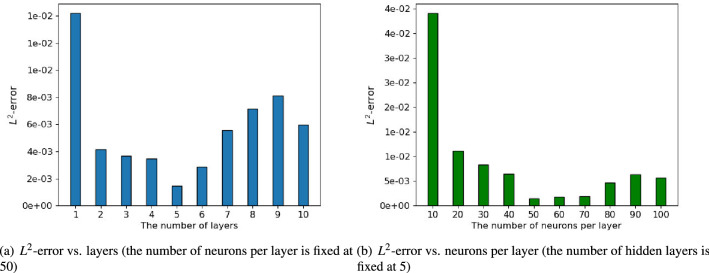
Performances of different architectural designs obtained by varying the number of hidden layers and the number of neurons per layer.

In this case, we also investigate the effect of different architectures, i.e., the number of hidden layers and the number of neurons per layer, on the relative $L^2$-error of the predicted solution. Figure 6a shows the performance when the number of model layers varies from 1 to 10. As we can see, a single−layer architecture tends to return incorrect predictions. By increasing the number of layers, the improved surrogate model yields a better accuracy. However, we can also observe that excessive layers will lead to overfitting of the model, resulting in suboptimal results. Similar results can be obtained in Fig. 6b, which analyzes the performance for a varying number of neurons. It can be seen that the increase in the number of neurons may not guarantee the desired performance, and the model works best when the number of neurons per layer is 50 to 70.

### Lid-driven cavity flow

**Figure 7:**
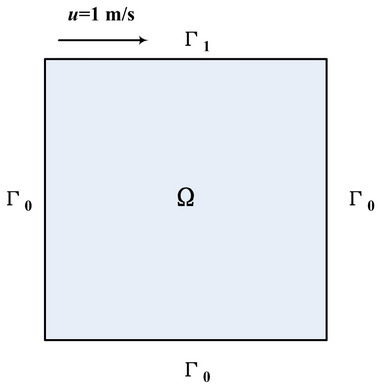
Lid-driven cavity flow.

In the last case, we employ a canonical benchmark problem, the steady-state flow in a two-dimensional lid-driven cavity (see Fig. 7), to analyze the performance of the DNN-based surrogate models. The flow system is governed by the Navier–Stokes equation^42,43^, which can be written as:

26 $$\begin{aligned} u(x,y) \cdot \nabla u(x,y) +\nabla p(x,y) -\frac{1}{Re} \Delta u(x,y)=0 \quad (x,y) \in \Omega , \end{aligned}$$

27 $$\begin{aligned} \nabla \cdot u(x,y)=0 \quad \quad (x,y) \in \Omega , \end{aligned}$$

28 $$\begin{aligned} u(x,y)=(1,0) \quad \quad (x,y) \in \Gamma _1, \end{aligned}$$

29 $$\begin{aligned} u(x,y)=(0,0) \quad \quad (x,y) \in \Gamma _0, \end{aligned}$$

where *u*(*x*, *y*) is a velocity vector field, *p*(*x*, *y*) is a scalar pressure field, *Re* is the Reynolds number of the flow, $\Omega \in [0,1]\times [0,1]$, $\Gamma _1$ denotes the top boundary of the two-dimensional square cavity, and $\Gamma _0$ denotes the other three sides.

In our experiments, we perform a neural network-based simulation using the vorticity–velocity (VV) formulation of the Navier–Stokes equations^42^. In this formulation, the velocity components *u*, *v* are obtained by taking derivatives of the scalar potential function $\psi (x, y)$ with respect to the *x* and *y* coordinates:

30 $$\begin{aligned} u=\frac{\partial \psi (x, y)}{\partial y}, \quad \quad v=-\frac{\partial \psi (x, y)}{\partial x}. \end{aligned}$$

As a result, the continuity equation $\nabla \cdot u(x,y)=0$ for incompressible fluids is automatically satisfied. Moreover, since only steady-state solutions are considered for this proof of concept, the constraint of temporal terms can be neglected. The corresponding loss function for this benchmark is defined as:

31 $$\begin{aligned} \begin{aligned} Loss(\theta _{W,b},x,y) \;&=\; \frac{1}{N_r}\sum ^{N_r}_{n=1}\omega _n \cdot |u \frac{\partial u}{\partial x}+v \frac{\partial u}{\partial y}+\frac{\partial p}{\partial x}-\frac{1}{R e}\left( \frac{\partial ^{2} u}{\partial x^{2}}+\frac{\partial ^{2} u}{\partial y^{2}}\right) |^2 \\& \quad + \frac{1}{N_r}\sum ^{N_r}_{n=1}\omega _n \cdot |u \frac{\partial v}{\partial x}+v \frac{\partial v}{\partial y}+\frac{\partial p}{\partial y}-\frac{1}{R e}\left( \frac{\partial ^{2} v}{\partial x^{2}}+\frac{\partial ^{2} v}{\partial y^{2}}\right) |^2 \\&\quad + \frac{1}{N_b}\sum ^{N_b}_{n=1}\omega _n \cdot |u-h_1(x,y)|^2 + \frac{1}{N_b}\sum ^{N_b}_{n=1}\omega _n \cdot |v-h_0(x,y)|^2, \end{aligned} \end{aligned}$$

where $h_1(x,y)=(1,0)$ for $(x,y) \in \Gamma _1$, and $h_0(x,y)=(0,0)$ for $(x,y) \in \Gamma _0$. The first and second derivative terms of $\psi$, *u*, *v*, and *p* with respect to the spatial coordinates (*x*, *t*) are computed using automatic differentiation.

In this case, we conduct experiments at Reynolds numbers $Re=100, 300, 600$ to comprehensively study the prediction performance of neural network-based surrogate models. The DFS-Net we used again contains three hidden layers. To better infer the PDE solutions, we gradually increase the number of neurons in each hidden layer as the Reynolds number increases: 50 neurons per layer at $Re=100$, 128 at $Re=300$, and 256 at $Re=600$. For each *Re*, we implement two modes of DFS-Net for training by means of the point weighting mechanism and excitation blocks. The models take the expanded spatial coordinates as inputs and output the pressure and vorticity fields. To validate the prediction accuracy of the trained models, we solve the Navier–Stokes equations to generate the reference solution using the open-source CFD solver OpenFOAM^44^.

**Figure 8:**
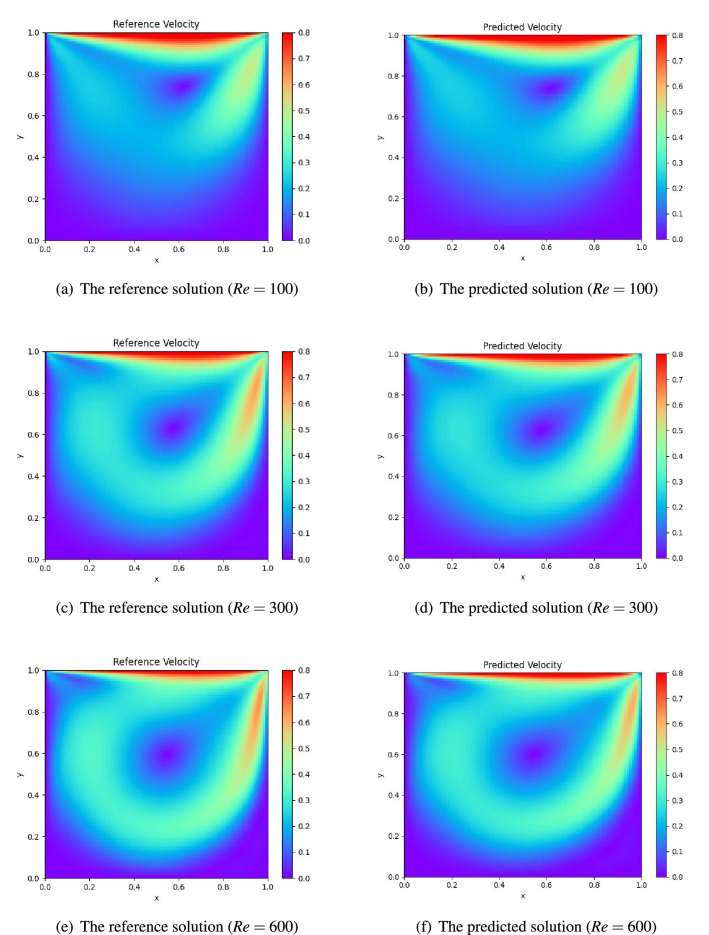
Lid-driven cavity flow: comparison of the reference solution with the predicted solution given by DFS-Net.

For different Reynolds numbers, DFS-Nets show a rapid convergence and reach their local optimum after approximately 20,000 epochs. The reference and predicted distributions of velocity are shown in Fig. 8. It is observed that DFS-Net can accurately capture the intricate nonlinear behavior of the Navier–Stokes equations and agrees well with the CFD solutions.

**Figure 9:**
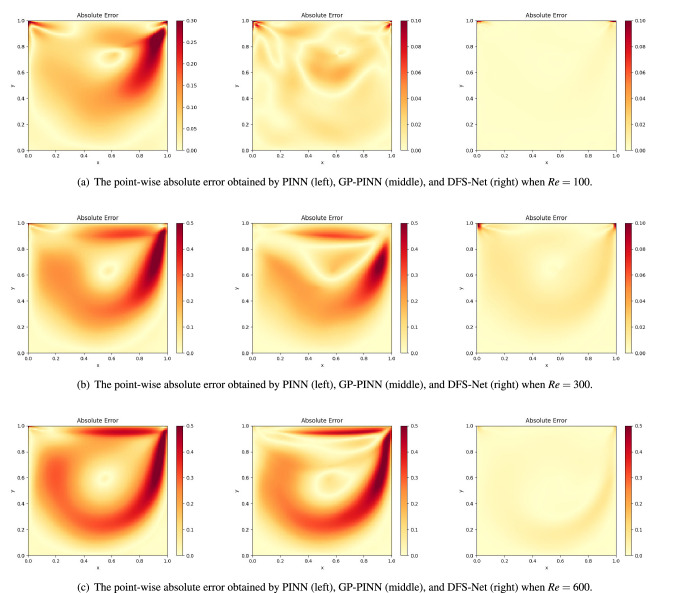
Performance of different surrogate models on the lid-driven cavity flow benchmarks.

It is worth noting that in this case, the velocity of *u* changes sharply from 0 to 1 at the junction of $\Gamma _1$ and $\Gamma _0$ [e.g., points (0,1) and (1,1)] according to the definition of the boundary velocity. These sharp discontinuities can lead to instability during neural network training in the near-wall regions. To emphasize the ability of the proposed model to handle nonlinearity in different subdomains, we plot and compare the point-wise absolute error obtained by PINN, GP-PINN, and DFS-Net in Fig. 9. It is observed that PINN fails to provide satisfactory prediction results for the underlying NS equations with different *Re* settings. At a Reynolds number of 100, PINN suffers a large error near the right boundary, yielding a prediction error of 3.47e−01. As the Reynolds number increases, the errors are more severe: 6.25e−01 at $Re=300$ and 7.76e−01 at $Re=600$.

When $Re=100$, GP-PINN can mitigate the prediction errors caused by sharp discontinuities. However, the incorrect prediction in the near-wall subdomains is still obvious (see Fig. 9). In contrast, the proposed DFS-Net appears to be more robust as the Reynolds number increases. The visualization results show that DFS-Net has a better ability to approximate complicated functions than other models and obtains a stable prediction accuracy in all three *Re* cases. This proves that the combined use of the weighting mechanism and the excitation blocks in DFS-Net has a positive effect on the velocity prediction of near-wall regions. The weighting mechanism assigns different weights to the sample points, thus eliminating any potential discontinuities (the loss weight of the discontinuous points is 0). Meanwhile, the introduced excitation blocks work as a tool to bias the allocation of available processing resources towards the most informative components of the expanded channels and correspondingly increase the weights of these solution-related features. As a result, they speed up the convergence of DFS-Net and allow us to achieve better accuracy.

To further analyze the performance of our method, we compare the $L^2$-error given by DFS-Nets against the other four widely used surrogate models. The experimental results are summarized in Tables 4, 5 and 6. When $Re=100$, DFS-Net$_{fix}$ and DFS-Net$_{unfix}$ perform better than the other comparative models, and the resulting prediction error is measured at 1.34e−02 and 2.91e−02 in the relative $L^2$-error, respectively. These two models improve the prediction accuracy of PINN and PINN-anneal by a factor of 5-25, although more training time is required. Compared with DGM and GP-PINN, the proposed models are more accurate and more efficient. Benefiting from the lightweight structure of DFS-Nets, the total training time of DFS-Net$_{fix}$ is 1610.1 s, while that of DFS-Net$_{unfix}$ is 1730.3 s. The prediction overhead for each sample is approximately 0.5 ms.

When $Re=300$ and 600, DFS-Net uniformly leads to the most accurate results we have obtained for this benchmark. Compared with the widely used models, the prediction error of the two DFS-Nets can be largely reduced by nearly an order of magnitude, yielding $L^2$-errors ranging from 3.31–3.80% at $Re=$300 and 7.25–8.50% at $Re=$600. We also conducted experiments for higher *Re* settings to verify the performance of the proposed methodology. However, problems with higher Reynolds numbers tend to have more stringent requirements on the depth (width) of the underlying network, which can lead to very large training overheads (e.g., more than 100k epochs of training are required to ensure a $L^2$-error of less than 10% at a Reynolds number of 1000). The excessive training overhead weakens the practicability of neural network-based methods and is therefore not discussed in this paper.

**Table 4:** Comparison of the relative $L^2$-error of different neural network-based surrogate models on the lid-driven cavity flow benchmark ($Re=100$).

Surrogate model^a^	Accuracy ($L^2$-error)	Training time (ms)^b^
DGM	6.69e−02	275.39
PINN	3.47e−01	13.33
PINN-anneal	1.42e−01	15.44
GP-PINN	4.01e−02	66.07
DFS-Net$_{fix}$	2.91e−02	58.68^c^
DFS-Net$_{unfix}$	1.34e−02	63.53^d^

^a^All models consists of three hidden layers with 50 neurons in each layer.

^b^The training time for each Adam epoch.

^c^The training time of DFS-Net$_{fix}$ for each L-BFGS-B epoch is 103.16 ms.

^d^ The training time of DFS-Net$_{unfix}$ for each L-BFGS-B epoch is 110.38 ms.

**Table 5:** Comparison of the relative $L^2$-error of different neural network-based surrogate models on the lid-driven cavity flow benchmark ($Re=300$).

Surrogate model^a^	Accuracy ($L^2$-error)	Training time (ms)^b^
DGM	5.96e−01	309.31
PINN	6.28e−01	15.11
PINN-anneal	6.17e−01	17.46
GP-PINN	4.47e−01	74.65
DFS-Net$_{fix}$	3.80e−02	66.63^c^
DFS-Net$_{unfix}$	3.31e−02	71.80^d^

^a^All models consists of three hidden layers with 128 neurons in each layer.

^b^The training time for each Adam epoch.

^c^The training time of DFS-Net$_{fix}$ for each L-BFGS-B epoch is 280.56 ms.

^d^The training time of DFS-Net$_{unfix}$ for each L-BFGS-B epoch is 297.83 ms.

**Table 6:** Comparison of the relative $L^2$-error of different neural network-based surrogate models on the lid-driven cavity flow benchmark ($Re=600$).

Surrogate model^a^	Accuracy ($L^2$-error)	Training time (ms)^b^
DGM	5.74e−01	410.30
PINN	7.76e−01	19.53
PINN-anneal	6.99e−01	23.14
GP-PINN	5.92e−01	96.25
DFS-Net$_{fix}$	8.50e−02	88.09^c^
DFS-Net$_{unfix}$	7.25e−02	92.83^d^

^a^All models consists of three hidden layers with 256 neurons in each layer.

^b^The training time for each Adam epoch.

^c^The training time of DFS-Net$_{fix}$ for each L-BFGS-B epoch is 364.33 ms.

^d^The training time of DFS-Net$_{unfix}$ for each L-BFGS-B epoch is 395.17 ms.

**Figure 10:**
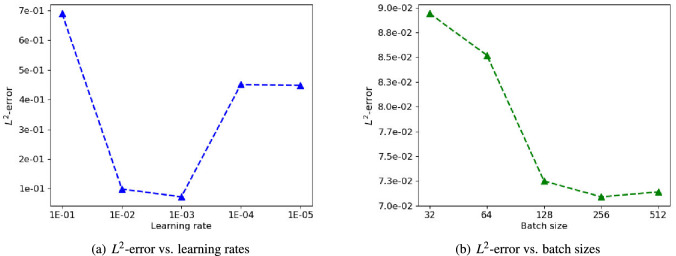
A comparison of the $L^2$-error for different learning rates and batch sizes.

The tuning of hyperparameters is an essential ingredient and important process of deep learning methods. Here, we evaluate the effect of two basic training hyperparameters, namely, the learning rate and batch size, at $Re=600$. During Adam-based training, the learning rate is a key hyperparameter used to control the step size of the gradient descent. An overly large learning rate might overshoot and prevent convergence, while at small step size may get stuck in a local minima, thus providing suboptimal solutions. A comparison of the $L^2$-error for different learning rates is shown in Fig. 10a. We can see that in this test case, the range of 1.0e−02 to 1.0e−03 yields a good convergence. Figure 10b depicts the performance when DFS-Net is trained on different batch sizes. Due to the memory limitations of the underlying system, we only test the results for batch sizes smaller than 512. The experimental results show that a relatively large batch size is required in order to achieve the desired accuracy, and that the minimum error is achieved when the batch size is 256.

Overall, we performed a comprehensive study on the robustness of the proposed DFS-Net model using three PDE benchmarks (Helmholtz equation, Klein–Gordon equation, and Navier–Stokes equation). To keep the training and prediction costs low, we did not consider very deep architectures throughout all test cases. Instead, we employed a fixed neural architecture (less than 256 neural units per layer) to evaluate the $L^2$-error against the reference solution, as well as the time cost required to complete each simulation. The experimental results demonstrate that DFS-Net is able to alleviate the problem of unstable predictions of existing neural network-based surrogate models and infer a solution of the underlying partial differential equations with a remarkable accuracy.

## Conclusion

Deep neural networks provide an efficient substitute for inferring PDE solutions because of their universal approximation capabilities in the high-dimensional parameter space. In this paper, we designed an improved neural network-based surrogate model, DFS-Net, for PDE solving. The proposed model employs a series of attention-based neural units to approximate the nonlinear mapping relations between the coordinate inputs and predictions. Moreover, we introduced a weighting mechanism in DFS-Net to enhance its ability to encode the underlying physical laws that govern a given PDE system. After suitable training, DFS-Net allows us to construct a computationally efficient and fully differentiable surrogate, where the quantities of interest can be immediately obtained by evaluating the trained network with any given input point without meshing.

To verify the robustness of DFS-Net, we conducted a collection of numerical studies on different surrogate models in terms of their learning efficiency and prediction accuracy. The experiments demonstrated that DFS-Net is able to yield a good trade-off between accuracy and efficiency. It outperforms the widely used surrogate models and achieves the best prediction performance on different numerical benchmarks, including the Helmholtz, Klein–Gordon, and Navier–Stokes equations.

Designing a suitable deep neural network for the PDE system is challenging, despite there are many architectural and parametric possibilities to consider. In future work, we will focus on studying the implicitly encoded features in the current DFS-Net and calibrating the model to more complex tasks. As deep learning technology is continuing to grow rapidly in terms of both methodological and algorithmic developments, we believe that this new class of universal function approximators has high potential for data-efficient prediction, control, and optimization across a wide range of physical applications.

## References

[1] Chen X, Vaidya J, Li J (2018). TAMM: A new topology-aware mapping method for parallel applications on the Tianhe-2A supercomputer. Algorithms and Architectures for Parallel Processing.

[2] Jagtap AD, Kharazmi E, Karniadakis GE (2020). Conservative physics-informed neural networks on discrete domains for conservation laws: Applications to forward and inverse problems. Comput. Methods Appl. Mech. Eng..

[3] Pang G, Karniadakis GE (2020). Physics-Informed Learning Machines for Partial Differential Equations: Gaussian Processes Versus Neural Network.

[4] Anderson JD, Wendt J (1995). Computational Fluid Dynamics.

[5] Mishra S (2019). A machine learning framework for data driven acceleration of computations of differential equations. Math. Eng..

[6] Sun L, Gao H, Pan S, Wang J-X (2020). Surrogate modeling for fluid flows based on physics-constrained deep learning without simulation data. Comput. Methods Appl. Mech. Eng..

[7] Brink AR, Najera-Flores DA, Martinez C (2020). The neural network collocation method for solving partial differential equations. Neural Comput. Appl..

[8] Dwivedi V, Srinivasan B (2020). Physics informed extreme learning machine (PIELM): A rapid method for the numerical solution of partial differential equations. Neurocomputing.

[9] Chen X (2020). Developing a new mesh quality evaluation method based on convolutional neural network. Eng. Appl. Comput. Fluid Mech..

[10] Chen, X., Liu, J., Gong, C., Pang, Y. & Chen, B. An airfoil mesh quality criterion using deep neural networks. in *12th International Conference on Advanced Computational Intelligence*, 536–541 (2020).

[11] Raissi M, Perdikaris P, Karniadakis GE (2017). Machine learning of linear differential equations using Gaussian processes. J. Comput. Phys..

[12] Raissi M, Perdikaris P, Karniadakis GE (2018). Numerical Gaussian processes for time-dependent and nonlinear partial differential equations. SIAM J. Sci. Comput..

[13] Tartakovsky A, Barajas-Solano D, He Q (2021). Physics-informed machine learning with conditional Karhunen–Loève expansions. J. Comput. Phys..

[14] Ahalpara DP (2015). Sniffer technique for numerical solution of Korteweg–de Vries equation using genetic algorithm. J. Appl. Math. Phys..

[15] Wang J-X, Wu J-L, Xiao H (2017). Physics-informed machine learning approach for reconstructing Reynolds stress modeling discrepancies based on DNS data. Phys. Rev. Fluids.

[16] Yang L, Zhang D, Karniadakis GE (2020). Physics-informed generative adversarial networks for stochastic differential equations. SIAM J. Sci. Comput..

[17] Li J, Chen Y (2020). Solving second-order nonlinear evolution partial differential equations using deep learning. Eng. Appl. Comput. Fluid Mech..

[18] Li Y, Mei F (2020). Deep learning-based method coupled with small sample learning for solving partial differential equations. Multimed. Tools Appl..

[19] Pawar S, San O, Aksoylu B, Rasheed A, Kvamsdal T (2021). Physics guided machine learning using simplified theories. Phys. Fluids.

[20] Xu H, Zhang D, Zeng J (2021). Deep-learning of parametric partial differential equations from sparse and noisy data. Phys. Fluids.

[21] Chen T, Hong C (1995). Universal approximation to nonlinear operators by neural networks with arbitrary activation functions and its application to dynamical systems. IEEE Trans. Neural Netw..

[22] Lu, L., Jin, P. & Karniadakis, G. E. DeepONet: Learning nonlinear operators for identifying differential equations based on the universal approximation theorem of operators (2020). 1910.03193.

[23] Raissi M, Perdikaris P, Karniadakis G (2019). Physics-informed neural networks: A deep learning framework for solving forward and inverse problems involving nonlinear partial differential equations. J. Comput. Phys..

[24] Raissi M, Yazdani A, Karniadakis GE (2020). Hidden fluid mechanics: Learning velocity and pressure fields from flow visualizations. Science.

[25] Wang S, Teng Y, Perdikaris P (2020). Understanding and mitigating gradient pathologies in physics-informed. Neural Netw..

[26] Sirignano J, Spiliopoulos K (2018). DGM: A deep learning algorithm for solving partial differential equations. J. Comput. Phys..

[27] Reinbold PAK, Grigoriev RO (2019). Data-driven discovery of partial differential equation models with latent variables. Phys. Rev. E.

[28] Zhang Y, Zhu X, Gao J (2020). Parameter estimation of acoustic wave equations using hidden physics models. IEEE Trans. Geosci. Remote Sens..

[29] Wandel N, Weinmann M, Klein R (2021). Teaching the incompressible Navier–Stokes equations to fast neural surrogate models in three dimensions. Phys. Fluids.

[30] De Florio M, Schiassi E, Ganapol BD, Furfaro R (2021). Physics-informed neural networks for rarefied-gas dynamics: Thermal creep flow in the Bhatnagar–Gross0–Krook approximation. Phys. Fluids.

[31] Kharazmi E, Zhang Z, Karniadakis GE (2021). hp-VPINNs: Variational physics-informed neural networks with domain decomposition. Comput. Methods Appl. Mech. Eng..

[32] Fang Z (2021). A high-efficient hybrid physics-informed neural networks based on convolutional neural network. IEEE Trans. Neural Netw. Learn. Syst..

[33] Hu, J., Shen, L. & Sun, G. Squeeze-and-excitation networks. In *Proceedings of the IEEE Conference on Computer Vision and Pattern Recognition (CVPR)* (2018).

[34] Jagtap AD, Kawaguchi K, Karniadakis GE (2020). Adaptive activation functions accelerate convergence in deep and physics-informed neural networks. J. Comput. Phys..

[35] Ramachandran, P., Zoph, B. & Le, Q. V. Searching for activation functions. 1710.05941 (2017).

[36] Morales J, Nocedal J (2011). Remark on algorithm 778: L-BFGS-B: Fortran subroutines for large-scale bound constrained optimization. ACM Trans. Math. Softw..

[37] van der Walt S, Colbert SC, Varoquaux G (2011). The numpy array: A structure for efficient numerical computation. Comput. Sci. Eng..

[38] Baydin AG, Pearlmutter BA, Radul AA, Siskind JM (2017). Automatic differentiation in machine learning: A survey. J. Mach. Learn. Res..

[39] Abadi, M. *et al.* Tensorflow: A system for large-scale machine learning. In *12th USENIX Symposium on Operating Systems Design and Implementation (OSDI 16)*, 265–283 (USENIX Association, 2016).

[40] Babuska I, Ihlenburg F, Paik E, Sauter S (1995). A generalized finite element method for solving the Helmholtz equation in two dimensions with minimal pollution. Comput. Methods Appl. Mech. Eng..

[41] Li Q (2011). Numerical solution of nonlinear Klein–Gordon equation using lattice Boltzmann method. Appl. Math..

[42] Jin X, Cai S, Li H, Karniadakis GE (2021). NSFnets (Navier–Stokes flow nets): Physics-informed neural networks for the incompressible Navier–Stokes equations. J. Comput. Phys..

[43] Arthurs CJ, King AP (2021). Active training of physics-informed neural networks to aggregate and interpolate parametric solutions to the Navier–Stokes equations. J. Comput. Phys..

[44] Jasak, H., Jemcov, A. & Tukovic, Z. OpenFOAM: A C++ library for complex physics simulations. *In International Workshop on Coupled Methods in Numerical Dynamics* **1–20**, (2007).

